# Anticoagulant and Antiplatelet Therapy in Patients with Atrial Fibrillation and Coronary Artery Disease

**DOI:** 10.1155/2012/184573

**Published:** 2012-04-23

**Authors:** Karl Mischke, Christian Knackstedt, Nikolaus Marx

**Affiliations:** ^1^Department of Cardiology, Pneumology, Angiology and Intensive Care Medicine, University Hospital, RWTH Aachen University, 52072 Aachen, Germany; ^2^Department of Cardiology, Maastricht University Hospital, 6202 AZ Maastricht, The Netherlands

## Abstract

Anticoagulation represents the mainstay of therapy for most patients with atrial fibrillation. Patients on oral anticoagulation often require concomitant antiplatelet therapy, mostly because of coronary artery disease. After coronary stent implantation, dual antiplatelet therapy is necessary. However, the combination of oral anticoagulation and antiplatelet therapy increases the bleeding risk. Risk scores such as the CHA_2_DS_2_-Vasc score and the HAS-BLED score help to identify both bleeding and stroke risk in individual patients. The guidelines of the European Society of Cardiology provide a rather detailed recommendation for patients on oral anticoagulation after coronary stent implantation. However, robust evidence is lacking for some of the recommendations, and especially for new oral anticoagulants and new antiplatelets few or no data are available. This review addresses some of the critical points of the guidelines and discusses potential advantages of new anticoagulants in patients with atrial fibrillation after stent implantation.

## 1. Introduction

Both patients with coronary artery disease as well as patients with atrial fibrillation frequently require antiplatelet therapy or anticoagulation to reduce the risk of cardiovascular and cerebral events. Recently, a number of new antiplatelet drugs and new drugs for anticoagulation have been approved to expand the armory of the treating physician. The standard antithrombotic agent for patients with coronary artery disease is aspirin; usually a dual therapy with addition of clopidogrel or newer agents is required after stent implantation or acute coronary syndrome. In patients with chronic atrial fibrillation, oral anticoagulation with vitamin K-antagonists represents the mainstay of therapy. In patients presenting with both coronary artery disease and atrial fibrillation, the choice of medication is sometimes challenging, especially with respect to a possible increase in bleeding complications in patients treated with a dual or triple therapy.

## 2. Risk Assessment in Atrial Fibrillation

The current guidelines of the European Society of Cardiology (ESC) for atrial fibrillation suggest risk stratification in patients with nonvalvular atrial fibrillation for the decision of implementing oral anticoagulation [1]. This risk stratification is based on the CHADS_2_- and CHA_2_DS_2_-Vasc-Score [1]. The CHA_2_DS_2_-Vasc-Score is depicted in Table 1. These scores can easily be implemented into clinical routine as they are simple, although they do not take into account other risk factors such as left atrial flow velocity or the different odds ratios of the included risk factors, with exception of a higher weight of previous stroke and high age (attribution of 2 points).

Numerous studies have demonstrated the superiority of oral anticoagulation compared to antiplatelet therapy in patients with atrial fibrillation and an increased risk for cerebrovascular events [2]. Thus, in patients with major risk factors, such as previous stroke, and in patients with two or more nonmajor risk factors, oral anticoagulation is recommended [1]. In patients with only one nonmajor risk factor, oral anticoagulation is also the preferred therapy; alternatively aspirin (75–325 mg/d) should be used. Thus, the majority of patients with atrial fibrillation requires oral anticoagulation [3, 4]. In patients without risk factors, the preferred treatment is no antithrombotic therapy rather than aspirin [1]. However, anticoagulation with vitamin K-antagonists is widely underused in clinical practise, partly due to disadvantages of vitamin-K-antagonists such as cumbersome INR-monitoring or drug interactions [5].

Anticoagulation is associated with an increased risk of bleeding. The ESC guidelines on atrial fibrillation also provide a risk score to assess the haemorrhagic risk: the HAS-BLED-Score (Table 2) [1]. However, some factors, for example, increased age, are associated with an increased risk for stroke as well as an increased risk for bleeding.

## 3. Antithrombotic Therapy after Stent Implantation in Patients with Atrial Fibrillation

A practical approach to the management of patients with atrial fibrillation who need oral anticoagulation after coronary artery stenting is presented in the current ESC guidelines for atrial fibrillation [1]. These recommendations are depicted in Table 3 for patients with a low or intermediate risk of bleeding and in Table 4 for patients with a high risk of bleeding. Basically, a triple therapy consisting of a vitamin-K-antagonist, aspirin, and clopidogrel is recommended for a short period of time and a vitamin-K-antagonist alone as lifelong therapy. Depending on the clinical presentation (acute coronary syndrome versus elective stenting), the hemorrhagic risk and the type of stent used (drug eluting versus bare metal stent), the recommended duration of triple therapy varies between 2 weeks and 6 months, and a dual therapy consisting of a vitamin-K-antagonist and clopidogrel might be used after the triple therapy for up to 12 months after stenting.

These recommendations can be used as a “roadmap” for the management of patients with atrial fibrillation and coronary stenting especially because they are detailed and pragmatic. However, these recommendations are based mainly on small studies, retrospective analyses, or expert opinion.

A different, less detailed decision algorithm has been suggested by Paikin et al. in Circulation [6]. In contrast to the ESC guidelines, oral anticoagulation is temporarily withheld in patients with a high risk of bleeding and a high thromboembolic risk (Table 5). According to this algorithm, triple therapy is primarily used in patients with a high thromboembolic and low bleeding risk. In conclusion, this algorithm is thus less aggressive in anticoagulation therapy than the ESC suggestion.

In a large Danish registry including more than 40.000 patients with myocardial infarction, yearly bleeding rates in patients receiving triple therapy (aspirin + clopidogrel + vitamin K antagonist) or dual therapy consisting of clopidogrel and vitamin K antagonist were 12% [7]. In contrast, bleeding rates for monotherapy (aspirin, clopidogrel, or vitamin K antagonist) and dual therapy with aspirin and vitamin K antagonist were 3–5%. These results were confirmed by Danish registries including more than 130.000 patients with atrial fibrillation: the combination of aspirin and vitamin K antagonists significantly increased the bleeding risk without yielding an additional benefit for stroke reduction compared with vitamin K antagonists only [8]. Bleeding leads to an increase in cardiovascular events and death, and this interaction is complex and attributable to several reasons: for instance, antiplatelet therapy is more likely to be withdrawn in patients with bleeding complications, thereby increasing the risk of subsequent cardiovascular events, blood transfusions might be directly harmful, and an increase in immature platelets might contribute to a prothrombotic state [9]. In addition, the variable pharmacodynamic response to clopidogrel and narrow therapeutic window of vitamin K antagonists are relevant disadvantages which might be reduced in new drugs with a more predictable level of P2Y12 inhibition or anticoagulation [9].

Several prospective studies are currently conducted to assess the use of dual and triple therapy in patients with atrial fibrillation after stent implantation. The MUSICA-2 trial (NCT01141153) compares dual antiplatelet therapy (aspirin 300 mg/day + clopidogrel 75 mg/day) with a triple regimen (Acenocoumarol + aspirin 100 mg/day + Clopidogrel 75 mg/day) in patients with atrial fibrillation and a low-to-moderate risk of stroke (CHADS ≤ 2) who are undergoing coronary stent implantation. The study is estimated to be completed in December 2012 with about 300 enrolled patients [10]. The ISAR Triple trial (NCT00776633) examines 600 patients on oral anticoagulation who undergo drug eluting stent implantation with concomitant aspirin and clopidogrel medication for either a short duration of 6 weeks or a longer duration of 6 months; the study is estimated to be completed in July 2012.

In addition, further issues to reduce both thromboembolic as well as bleeding complications (such as radial versus femoral access site) are addressed in a consensus document of the European Society of Cardiology Working Group on Thrombosis and the European Association of Percutaneous Cardiovascular Interventions [11].

In clinical practise, especially two aspects of the ESC guidelines for atrial fibrillation might pose a challenge to physicians. (1) Oral anticoagulation is recommended for the majority of patients with atrial fibrillation, and especially a triple therapy with concomitant antiplatelets dramatically increases the risk of bleeding in individual patients. On the other hand, dual antiplatelet therapy after stent implantation is necessary to reduce the risk of an often fatal stent thrombosis. (2) Lifelong oral anticoagulation without concomitant antiplatelet therapy is recommended one year after stent implantation after acute coronary syndrome. Because of an annual rate of late stent thrombosis of about 0.6% even with antiplatelet therapy and the limitations of vitamin K antagonists to prevent stent thrombosis, a cautious approach to complete termination of antiplatelet therapy seems warranted [12, 13]. The American College of Chest Physicians guidelines from 2008 recommend the combined use of aspirin and vitamin K antagonists in patients after coronary stent implantation and an indication for oral anticoagulation as long-term treatment [14]. Although numerous studies have demonstrated the benefit of vitamin K antagonists in coronary artery disease and the ESC guidelines on ST-elevation myocardial infarctions from 2008 approve the replacement of aspirin by a vitamin K antagonist in patients with atrial fibrillation, most studies were performed before the use of drug eluting stents [15]. The ESC guidelines on myocardial revascularisation from 2010 do not address this topic.

The duration of clopidogrel after stent implantation has also been much debated. Although the use of clopidogrel for 6–12 months after drug eluting stent implantation is long enough for most patients, an observational study points at a possible benefit of an extended use of clopidogrel for more than 12 months in patients receiving a drug eluting stent [16]. The recently published EXCELLENT trial (Six-Month versus Twelve-Month Dual Antiplatelet Therapy after Implantation of Drug-Eluting Stents) demonstrated a superiority of 12 months of dual antiplatelet therapy compared with 6 months in a subgroup of diabetic patients [17]. Currently, the PRODIGY study looks at the use of clopidogrel for 6 or 24 months in a broad all-comer patient population receiving bare metal and drug eluting stents [18].

The ACTIVE-A trial (effect of clopidogrel added to aspirin in patients with atrial fibrillation) assessed the combined therapy of aspirin and clopidogrel in patients with atrial fibrillation who were considered unsuitable for therapy with vitamin K antagonists [19]. Patients on the combined therapy with clopidogrel and aspirin suffered significantly less often from stroke, albeit at the cost of increased bleeding rates. These results were considered in the 2011 update on the American guidelines (ACCF, AHA, and HRS) on atrial fibrillation, resulting in a class IIb recommendation for the combined use of clopidogrel and aspirin in patients who are considered unsuitable for vitamin K antagonist therapy [20]. However, the combination of aspirin and clopidogrel does poorly when compared to warfarin: the ACTIVE-W trial (atrial fibrillation clopidogrel trial with irbesartan for prevention of vascular events) compared clopidogrel plus aspirin with oral anticoagulation with warfarin in patients with atrial fibrillation and an increased risk for stroke. This trial demonstrated a reduction in vascular events in patients on warfarin without an increase in bleeding complications [21].

Many components of the CHA_2_DS_2_-Vasc-Score are also risk factors for atherosclerosis, and a high CHA_2_DS_2_-Vasc-Score can identify both patients with a high risk for stroke as well as patients with a high risk for ischemic heart disease [15]. Antiplatelet therapy or anticoagulation is thus often warranted both because of an increased stroke risk as well as an increased risk for cardiac events. Vitamin K antagonists may not provide a sufficient protection for cardiac and embolic events in patients who are not within the therapeutic range. One could speculate that this problem might be diminished by the use of new drugs for oral anticoagulation, for instance, dabigatran, rivaroxaban, or apixaban. Dabigatran is a direct thrombin inhibitor and rivaroxaban and apixaban inhibit factor Xa. However, in the RELY trial, a nonsignificant increase in myocardial infarction was observed in patients receiving dabigatran compared to warfarin [22].

So current ESC guidelines might lead to an increased risk of late stent thrombosis and insufficient protection of cardiac events in patients with poor INR control. In addition, the use of dabigatran as monotherapy might not be a good alternative in these patients. In contrast, fewer patients receiving apixaban had a myocardial infarction than those receiving warfarin in the ARISTOTLE study or aspirin in the AVERROES trial [23, 24]. In the ROCKET-AF study, a statistically not significant lower rate of myocardial infarction was observed in patients receiving rivaroxaban compared to placebo [25]. Thus, the use of apixaban or rivaroxaban or a combination of low-dose dabigatran and aspirin might be a good long-term alternative to vitamin K antagonists in patients with atrial fibrillation after stent implantation.

## 4. New Drugs for Oral Anticoagulation

After decades of research novel agents for oral anticoagulation have finally been introduced into the market. New drugs for oral anticoagulation include the direct thrombin inhibitor dabigatran etexilate as well as the factor Xa inhibitors rivaroxaban, apixaban, and edoxaban. The direct thrombin inhibitor ximelagatran was only available for a short period of time and taken from the market due to liver toxicity. Vitamin K antagonists interfere with the biosyntheses of coagulation factors and thus achieve effective levels of anticoagulation only after several days, and their effect diminishes slowly after withdrawal. In contrast, direct thrombin inhibitors and factor Xa inhibitors provide a sufficient anticoagulant effect within a few hours and their effects diminish fast. Further essentials of the new oral anticoagulants include a wide therapeutic window, little interaction with food intake, and other drugs compared to vitamin K antagonists. The cumbersome monitoring of the anticoagulation effect associated with vitamin K antagonists is thus not necessary any more in patients receiving these new substances. The problems of dosing of vitamin K antagonists result in a high percentage of inadequate anticoagulation levels: even in the setting of clinical trials, the overall time within in the therapeutic range is often <70%. Possible disadvantages of the new substances include the lack of antidotes, difficulties in effect monitoring, and the short half-life that might pose a problem in patients with low compliance. Recently, several large studies have demonstrated a benefit of the new oral anticoagulants compared to warfarin in patients with atrial fibrillation.

The RELY study included more than 18.000 patients with atrial fibrillation and a mean CHADS_2_-score of 2.1 randomized to dabigatran 110 mg bid, dabigatran 150 mg bid, or warfarin [22]. The high dose of dabigatran led to a significant decrease in stroke and systemic embolism without an increase in major hemorrhage. Mortality was also reduced, although not reaching statistical significance. Dabigatran applied in a low dose resulted in a significant decrease in major hemorrhage without an increase in ischemic stroke. The net clinical benefit (consisting of stroke and systemic embolism, major hemorrhage and mortality) was best for the group of patients with dabigatran 150 mg bid. However, fatal bleedings especially in older patients and in patients with an impaired renal function have been reported in patients receiving dabigatran. Indeed, a recent subgroup analysis of the RELY trial showed that patients ≥ 75 years had a similar or higher bleeding risk on dabigatran compared with warfarin, and renal function should be assessed yearly in patients ≥ 75 years as well as in clinical situations with a possible decline in renal function [26].

In the ARISTOTLE study, also more than 18.000 patients with atrial fibrillation and a mean CHADS_2_-score of 2.1 were included [23]. The study demonstrated a superiority of apixaban versus warfarin with a reduction in stroke and systemic embolism. In addition, apixaban caused less bleeding and resulted in a lower mortality.

The ROCKET-AF study included more than 14.000 patients with atrial fibrillation and demonstrated a noninferiority of the factor Xa inhibitor rivaroxaban compared to warfarin for the prevention of stroke or systemic embolism [25]. There was no difference in the risk of major bleeding, although fatal and intracranial bleeding occurred less frequently in the rivaroxaban group. In contrast to the RELY and ARISTOTLE study, a superiority of the new drug in the primary endpoint was not seen in the intention-to-treat population but only in the on-treatment population, although patients in the ROCKET-AF study had a higher mean CHADS_2_ score of 3.5 resulting in higher event rates than patients in the RELY and ARISTOTLE study. However, a potential benefit of rivaroxaban is the once-daily dose compared to the twice-daily intake of apixaban and dabigatran. In addition, a trend towards a higher risk of myocardial infarctions was seen in patients on dabigatran compared with warfarin, whereas this was not observed for rivaroxaban in the ROCKET-AF trial. Indeed, the ATLAS ACS 2-TIMI 51 demonstrated ad reduction in mortality in patients after acute coronary syndrome on low-dose rivaroxaban (2.5 mg bid) at the cost of higher bleeding complications [27].

**Figure 1:**
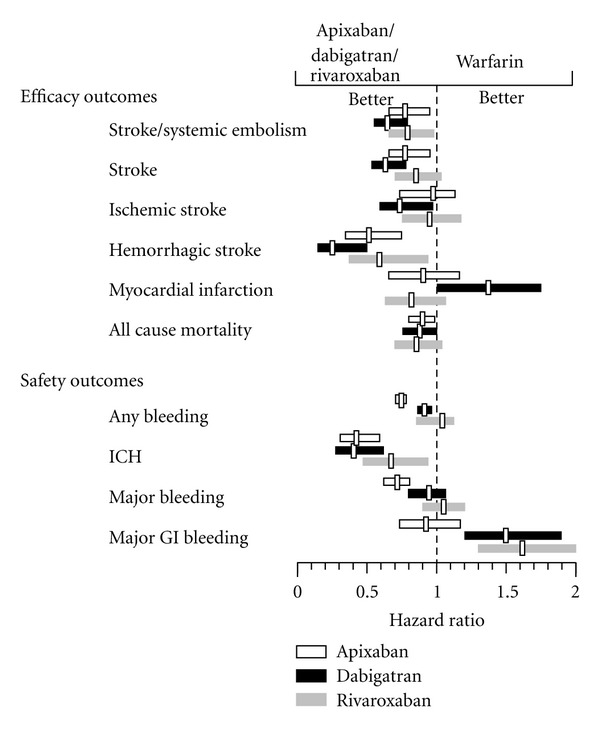
GI: gastrointestinal, ICH: intracerebral hemorrhage. Efficacy and safety outcomes in RELY, ARISTOTLE and ROCKET-AF [22, 23, 25]. Results are displayed for Dabigatran 150 mg bid.

The safety and efficacy outcomes of the RELY, ROCKET-AF, and ARISTOTLE study are depicted in Figure 1.

The oral factor Xa-inhibitor edoxaban is currently being tested in patients with atrial fibrillation in the Engage AF TIMI 48 study (NCT00781391) as well as in a Chinese study (NCT00806624). In addition, an international study with a short follow-up of 3 months is assessing bleeding complications (NCT00504556).

## 5. New Drugs for Oral Anticoagulation in Combination with Antiplatelet Therapy

The results of the three large studies RELY, ARISTOTLE, and ROCKET-AF are very promising. Concomitant use of antiplatelet therapy in patients with atrial fibrillation who need oral anticoagulation is common in clinical practice and was seen in about one third of the patients in these three studies. However, concomitant antiplatelet therapy in patients on warfarin due to atrial fibrillation increases the risk of hemorrhage: the combination of warfarin and aspirin is associated with an almost twofold bleeding risk and the combination of warfarin and clopidogrel or a triple therapy consisting of warfarin, aspirin, and clopidogrel with a threefold bleeding risk compared to warfarin monotherapy [28]. Data from prospective trials looking at the benefit and risk of combining oral factor Xa-inhibitors or direct thrombin inhibitors with antiplatelet therapy as a dual or triple therapy are missing. However, a post-hoc analysis from the RELY study has been published as an abstract, and a subgroup analysis is reported in the ARISTOTLE study.

In the ARISTOTLE study, 31% of the patient received concomitant aspirin medication and a low-percentage (1.9%) clopidogrel. In the subgroup analysis, there was no significant difference between patients receiving aspirin at randomization and those who did not with regard to stroke and major bleeding (*P* = 0.4 for interaction) [23]. 36% of the ROCKET-AF study population received concomitant aspirin therapy, up to now a subgroup analysis has not been published [25].

In the RELY study, almost 7000 patients (40% of the study population) received concomitant aspirin or clopidogrel. In accordance with the results of the main study, a post-hoc analysis showed that dabigatran 110 mg bid was noninferior to warfarin with regard to stroke and systemic embolism and superior to warfarin in terms of bleeding independent of a concomitant antiplatelet therapy. Dabigatran 150 mg bid was superior to warfarin with regard to stroke and systemic embolism, especially among patients without concomitant use of antiplatelet therapy (HR = 0.52, 95% CI: 0.38–0.72). This effect seemed to be reduced in patients with concomitant antiplatelet therapy (HR = 0.80; 95% CI: 0.59–1.08), although the difference was not statistically significant (*P* for interaction = 0.06). Dabigatran 150 mg bid was similar to warfarin in terms of bleeding complications regardless of concomitant antiplatelet therapy. The rates of major bleeding increased by 60% in patients on concomitant antiplatelet therapy (HR = 1.60, 95% CI = 1.41–1.81 after adjustment for age, gender, warfarin experience, systolic blood pressure, coronary artery disease, heart failure, hypertension, diabetes, prior TIA, creatinine clearance, and statin use) [29].

In conclusion, in patients who require dual or triple therapy, low-dose dabigatran or apixaban might be a good alternative to warfarin due to good efficacy and rather low bleeding complications. However, current data supporting this are scarce. A prospective randomized trial would be sensible but is unlikely to be conducted.

## 6. PAR-1 Receptor Antagonists

The protease-activated receptor-1 (PAR-1) thrombin receptor antagonists atopaxar and vorapaxar represent a novel approach to the inhibition of platelet activation. Thrombin can stimulate platelet activation via the PAR-1 receptor on the platelet surface, and PAR-1 thrombin receptor antagonists can thus reduce thrombin-induced platelet activation. In contrast, aspirin and P2Y12 receptor antagonists do not interfere with PAR-1-dependent platelet activation, and patients on dual antiplatelet therapy remain at risk of cardiovascular events because of alternative pathways of platelet activation [30].

The oral PAR-1 receptor antagonist vorapaxar is being evaluated in two phase III trials. However, the Trial to Assess the Effects of SCH 530348 in Preventing Heart Attack and Stroke in Patients with Acute Coronary Syndrome (TRACER) was stopped early; and vorapaxar was stopped in patients with a history of stroke in the Trial to Assess the Effects of SCH 530348 in Preventing Heart Attack and Stroke in Patients with Atherosclerosis (TRA 2°P-TIMI 50) due to an excess of intracranial hemorrhage [30, 31]. The trial is still ongoing for patients with a history of myocardial infarction or peripheral arterial disease.

The oral PAR-1-receptor antagonist Atopaxar has a shorter half-life than vorapaxar and has been tested in the phase II Lessons from Antagonizing the Cellular Effects of Thrombin (LANCELOT)-ACS trial. This trial demonstrated the safety and tolerability of atopaxar in patients with acute coronary syndrome and also displayed a trend towards a lower incidence of cardiovascular death, myocardial infarction, and stroke accompanied with a nonsignificant increase in the rate of bleeding [30]. This trial was conducted in parallel with the LANCELOT CAD trial, a phase II trial with patients with stable coronary artery disease [32]. In this trial, the use of atopaxar resulted in more minor bleeding and a trend towards fewer ischemic events.

Atopaxar might have a diminished risk of bleeding compared with traditional antithrombotic drugs as it selectively reduces thrombin-mediated platelet activation but does not disrupt thrombin-dependent fibrin generation or ADP-dependent platelet activation [30].

However, the efficacy and safety of atopaxar need to be proven in large phase III trials. With regard to vorapaxar, the excess rate in intracranial bleeding is sobering. No clinical data exist with regard to the combination of PAR-1 with warfarin or new anticoagulant drugs.

## 7. Further Strategies for Bleeding Risk Reduction

Several strategies apart from the choice of antiplatelet therapy and oral anticoagulation can reduce the risk of bleeding in patients with atrial fibrillation and coronary stenting. As triple therapy dramatically increases the bleeding risk, the duration of triple therapy should be limited to a minimum. Balloon angioplasty or the use of bare metal stents opposed to drug eluting stents can reduce the time of clopidogrel medication and should be preferred in patients requiring triple therapy [33]. In addition, during triple or dual therapy, the INR should be aimed at 2–2.5 rather than 2-3. The choice of access site for coronary interventions also has implications on the bleeding risk: radial access, which can also be performed in acute myocardial infarction, is associated with lower bleeding complications than a femoral access [34]. Peri-interventional antithrombotic therapy also affects bleeding complications, and favourable results have been observed for bivalirudin instead of unfractionated heparin in combination with a glycoprotein IIb/IIIa-inhibitor [35]. A proton pump inhibitor can reduce the risk of gastrointestinal bleeding in selected patients [36].

## 8. Conclusion

An individualized approach is needed for patients with atrial fibrillation and coronary artery disease to find the fine balance between the risk of cerebrovascular events and bleeding complications. Scores like the CHA_2_DS_2_-Vasc and the HAS-BLED score can help to define the need for oral anticoagulation and assess the bleeding risk in individual patients, and the ESC guidelines on atrial fibrillation provide a detailed suggestion for the combination of anticoagulation and antiplatelet therapy. However, these recommendations are mainly based on expert opinion and data derived from large prospective studies are lacking. The ESC guidelines on atrial fibrillation recommend a triple therapy consisting of aspirin, clopidogrel, and oral anticoagulation after stent implantation in patients with atrial fibrillation requiring oral anticoagulation. This poses patients at an increased risk for bleeding complications, and this problem might even be aggravated with the use of novel antiplatelets such as prasugrel or ticagrelor. On the other hand, monotherapy with oral anticoagulation is recommended for patients with stable coronary artery disease and atrial fibrillation; possibly increasing the risk of cardiac events.

New drugs for oral anticoagulation, especially apixaban and dabigatran, have shown convincing results in large clinical trials compared to warfarin. Although detailed data on patients with coronary artery disease receiving antiplatelets and novel anticoagulants are missing, especially the use of apixaban or low-dose dabigatran might be a good alternative to warfarin by possibly reducing the risk of bleeding (which is especially high in patients on triple therapy). In addition, especially apixaban—possibly in combination with aspirin—might also be a very good alternative to warfarin monotherapy in patients with stable coronary artery disease and atrial fibrillation. However, it has to be taken into account that data on the combination of aspirin and dabigatran or apixaban are all derived from subgroup analyses.

An individualized approach taking into account the individual risk of stroke, bleeding, myocardial infarction, and stent type is needed to assess the best treatment option, and hopefully new drugs will help to increase both efficacy and safety of the treatment.

## Figures and Tables

**Table 1:** CHA_2_DS_2_-Vasc-Score [1].

		Points
C	Congestive heart failure	1
H	Hypertension	1
A	Age ≥ 75 years	2
D	Diabetes mellitus	1
S	Stroke/TIA/thromboembolism	2
V	Vascular disease	1
A	Age 65–74 years	1
S	Sex category (female sex)	1

		max. 9

**Table 2:** HAS-BLED Score [1].

		Points
H	Hypertension	1
A	Abnormal renal and liver function (1 point each)	1 or 2
S	Stroke	1
B	Bleeding	1
L	Labile INRs	1
E	Elderly (e.g., ≥65 years)	1
D	Drugs or alcohol (1 point each)	1 or 2

		max. 9

Hypertension: systolic blood pressure > 160 mmHg. Abnormal kidney function: chronic dialysis or renal transplantation or creatinine ≥ 200 mmol/L. Abnormal liver function: chronic hepatic disease (e.g., cirrhosis) or biochemical evidence of significant hepatic derangement (e.g., bilirubin > 2x upper limit of normal, in association with aspartate aminotransferase/alanine aminotransferase/alkaline phosphatase > 3x upper limit normal, etc.). Bleeding: previous bleeding history and/or predisposition to bleeding, for example, bleeding diathesis, anaemia, and so forth. Labile INRs: unstable/high INRs or poor time in therapeutic range (e.g., <60%). Drugs/alcohol use: concomitant use of drugs, such as antiplatelet agents, nonsteroidal anti-inflammatory drugs, or alcohol abuse, and so forth INR: international normalized ratio.

**Table 3:** ESC Anticoagulation regimen in patients with low-intermediate bleeding risk after stent implantation [1].

Setting stent	Anticoagulation (HAS-BLED 0–2)
Elective BMS	1 month: VKA + aspirin + clopidogrel Lifelong: VKA
Elective DES	3* months: VKA + aspirin + clopidogrel Up to 12th month: VKA + clopidogrel (or aspirin) Lifelong: VKA
ACS BMS/DES	6 months: VKA + aspirin+clopidogrel Up to 12th month: VKA + clopidogrel (oraspirin) Lifelong: VKA

ACS: acute coronary syndrome, BMS: bare metal stent, DES: drug eluting stent, VKA: vitamin K-antagonist, *6 months in patients with a paclitaxel-eluting stent. The INR should be adjusted according to concomitant antiplatelet therapy (2-3 in vitamin K-antagonist monotherapy and 2–2.5 in case of concomitant antiplatelet therapy).

**Table 4:** ESC Anticoagulation regimen in patients with high bleeding risk after stent implantation [1].

Setting stent	Anticoagulation (HAS-BLED ≥ 3)
Elective BMS^#^	2–4 weeks: VKA + aspirin + clopidogrel Lifelong: VKA
ACS BMS^#^	4 weeks: VKA + aspirin+ clopidogrel Up to 12th month: VKA + clopidogrel (oraspirin) Lifelong: VKA

ACS: acute coronary syndrome, BMS: bare metal stent, DES: drug eluting stent, VKA: vitamin K-antagonist, ^#^DES should be avoided as far as possible; if used, triple therapy might be prolonged to 3–6 months. The INR should be adjusted according to concomitant antiplatelet therapy (2-3 in vitamin K-antagonist monotherapy and 2–2.5 in case of concomitant antiplatelet therapy).

**Table 5:** U. S. Anticoagulation regimen after stent implantation (adopted from Paikin et al.) [6].

Setting	Anticoagulation/Antiplatelets
CHADS_2 _0-1	Aspirin + clopidogrel
CHADS_2_ > 1, low bleeding risk	Aspirin + clopidogrel + warfarin
CHADS_2_ > 1, high bleeding risk	Aspirin + clopidogrel

CHADS_2_: cardiac failure, hypertension, age, diabetes, stroke (doubled). High risk of bleeding: for example, age > 75 year, severe renal dysfunction, recent gastrointestinal bleeding, prior stroke, uncontrolled hypertension. Bare metal stents should be preferred, the duration of triple therapy months should be restricted to 1 month after bare metal stent implantation and 3 after drug eluting stent implantation (6 months in paclitaxel stents).

## References

[1] Camm AJ, Kirchhof P, Lip GYH (2010). Guidelines for the management of atrial fibrillation: The Task Force for the Management of Atrial Fibrillation of the European Society of Cardiology (ESC). *European Heart Journal*.

[2] Hart RG, Benavente O, McBride R, Pearce LA (1999). Antithrombotic therapy to prevent stroke in patients with atrial fibrillation: a meta-analysis. *Annals of Internal Medicine*.

[3] Nabauer M, Gerth A, Limbourg T (2009). The registry of the German competence NETwork on atrial fibrillation: patient characteristics and initial management. *Europace*.

[4] Nieuwlaat R, Capucci A, Camm AJ (2005). Atrial fibrillation management: a prospective survey in ESC Member Countries: the Euro Heart Survey on atrial fibrillation. *European Heart Journal*.

[5] Gladstone DJ, Bui E, Fang J (2009). Potentially preventable strokes in high-risk patients with atrial fibrillation who are not adequately anticoagulated. *Stroke*.

[6] Paikin JS, Wright DS, Crowther MA, Mehta SR, Eikelboom JW (2010). Triple antithrombotic therapy in patients with atrial fibrillation and coronary artery stents. *Circulation*.

[7] Sørensen R, Hansen ML, Abildstrom SZ (2009). Risk of bleeding in patients with acute myocardial infarction treated with different combinations of aspirin, clopidogrel, and vitamin K antagonists in Denmark: a retrospective analysis of nationwide registry data. *The Lancet*.

[8] Olesen JB, Lip GYH, Lindhardsen J (2011). Risks of thromboembolism and bleeding with thromboprophylaxis in patients with atrial fibrillation: a net clinical benefit analysis using a “real world” nationwide cohort study. *Thrombosis and Haemostasis*.

[9] Grove EL, Storey RF (2009). The right oral antithrombotics in acute coronary syndromes. *The Lancet*.

[10] Dewilde W, Berg JT (2009). Design and rationale of the WOEST trial: what is the optimal antiplatelet and anticoagulant therapy in patients with oral anticoagulation and coronary StenTing (WOEST). *American Heart Journal*.

[11] Lip GYH, Huber K, Andreotti F (2010). Antithrombotic management of atrial fibrillation patients presenting with acute coronary syndrome and/or undergoing coronary stenting: executive summary. A Consensus Document of the European Society of Cardiology Working Group on Thrombosis, endorsed by the European Heart Rhythm Association (EHRA) and the European Association of Percutaneous Cardiovascular Interventions (EAPCI). *European Heart Journal*.

[12] Daemen J, Wenaweser P, Tsuchida K (2007). Early and late coronary stent thrombosis of sirolimus-eluting and paclitaxel-eluting stents in routine clinical practice: data from a large two-institutional cohort study. *Lancet*.

[13] Mischke K, Hoffmann R (2010). ESC guidelines on atrial fibrillation: impact on management of patients after stentimplantation. *European Heart Journal*.

[14] Becker RC, Meade TW, Berger PB (2008). The primary and secondary prevention of coronary artery disease: American College of Chest Physicians evidence-based clinical practice guidelines (8th edition). *Chest*.

[15] Van De Werf F, Bax J, Betriu A (2008). Management of acute myocardial infarction in patients presenting with persistent ST-segment elevation. *European Heart Journal*.

[16] Eisenstein EL, Anstrom KJ, Kong DF (2007). Clopidogrel use and long-term clinical outcomes after drug-eluting stent implantation. *Journal of the American Medical Association*.

[17] Gwon HC, Hahn JY, Park W (2012). Six-month versus 12-month dual antiplatelet therapy after implantation of drug-eluting stents: the efficacy of Xience/Promus versus cypher to reduce late loss after stenting (EXCELLENT) randomized, multicenter study. *Circulation*.

[18] Valgimigli M, Campo G, Percoco G (2010). Randomized comparison of 6-versus 24-month clopidogrel therapy after balancing anti-intimal hyperplasia stent potency in all-comer patients undergoing percutaneous coronary intervention: design and rationale for the PROlonging Dual-antiplatelet treatment after Grading stent-induced Intimal hyperplasia study (PRODIGY). *American Heart Journal*.

[19] Connolly SJ, Pogue J, Hart RG (2009). Effect of clopidogrel added to aspirin in patients with atrial fibrillation. *New England Journal of Medicine*.

[20] Wann LS, Curtis AB, January CT (2011). 2011 ACCF/AHA/HRS focused update on the management of patients with atrial fibrillation (Updating the 2006 Guideline): a report of the American college of cardiology foundation/American heart association task force on practice guidelines. *Circulation*.

[21] Connolly S, Pogue J, Hart R (2006). Clopidogrel plus aspirin versus oral anticoagulation for atrial fibrillation in the atrial fibrillation clopidogrel trial with irbesartan for prevention of vascular events (ACTIVE W): a randomised controlled trial. *Lancet*.

[22] Connolly SJ, Ezekowitz MD, Yusuf S (2009). Dabigatran versus warfarin in patients with atrial fibrillation. *New England Journal of Medicine*.

[23] Granger CB, Alexander JH, McMurray JJV (2011). Apixaban versus warfarin in patients with atrial fibrillation. *New England Journal of Medicine*.

[24] Connolly SJ, Eikelboom J, Joyner C (2011). Apixaban in patients with atrial fibrillation. *New England Journal of Medicine*.

[25] Patel MR, Mahaffey KW, Garg J (2011). Rivaroxaban versus warfarin in nonvalvular atrial fibrillation. *New England Journal of Medicine*.

[26] Eikelboom JW, Wallentin L, Connolly SJ (2011). Risk of bleeding with 2 doses of dabigatran compared with warfarin in older and younger patients with atrial fibrillation: an analysis of the randomized evaluation of long-term anticoagulant therapy (RE-LY) Trial. *Circulation*.

[27] Mega JL, Braunwald E, Wiviott SD (2012). Rivaroxaban in patients with a recent acute coronary syndrome. *New England Journal of Medicine*.

[28] Hansen ML, Sørensen R, Clausen MT (2010). Risk of bleeding with single, dual, or triple therapy with warfarin, aspirin, and clopidogrel in patients with atrial fibrillation. *Archives of Internal Medicine*.

[29] Dans AL, Connolly S, Brückmann M Concomitant use of antiplatelet therapy with dabigatran or warfarin in the randomized evaluation of long-term anticoagulation therapy (RE-LY) trial.

[30] O'Donoghue ML, Bhatt DL, Wiviott SD (2011). Safety and tolerability of atopaxar in the treatment of patients with acute coronary syndromes: the lessons from antagonizing the cellular effects of thrombin-acute coronary syndromes trial. *Circulation*.

[31] Morrow DA, Scirica BM, Fox KAA (2009). Evaluation of a novel antiplatelet agent for secondary prevention in patients with a history of atherosclerotic disease: design and rationale for the Thrombin-Receptor Antagonist in Secondary Prevention of Atherothrombotic Ischemic Events (TRA 2°P)-TIMI 50 trial. *American Heart Journal*.

[32] Wiviott SD, Flather MD, O'Donoghue ML (2011). Randomized trial of atopaxar in the treatment of patients with coronary artery disease: the lessons from antagonizing the cellular effect of thrombin-coronary artery disease trial. *Circulation*.

[33] Steg PG, Huber K, Andreotti F (2011). Bleeding in acute coronary syndromes and percutaneous coronary interventions: position paper by the Working Group on Thrombosis of the European Society of Cardiology. *European Heart Journal*.

[34] Jolly SS, Yusuf S, Cairns J (2011). Radial versus femoral access for coronary angiography and intervention in patients with acute coronary syndromes (RIVAL): a randomised, parallel group, multicentre trial. *The Lancet*.

[35] Stone GW, Witzenbichler B, Guagliumi G (2008). Bivalirudin during primary PCI in acute myocardial infarction. *New England Journal of Medicine*.

[36] Abraham NS, Hlatky MA, Antman EM (2010). ACCF/ACG/AHA 2010 expert consensus document on the concomitant use of proton pump inhibitors and thienopyridines: a focused update of the ACCF/ACG/AHA 2008 expert consensus document on reducing the gastrointestinal risks of antiplatelet therapy and NSAID use: a report of the American College of Cardiology Foundation Task Force on Expert Consensus Documents. *Circulation*.

